# A novel mechanism for the protection of embryonic stem cell derived tenocytes from inflammatory cytokine interleukin 1 beta

**DOI:** 10.1038/s41598-019-39370-4

**Published:** 2019-02-26

**Authors:** Alyce McClellan, Richard Evans, Cheryl Sze, Shohei Kan, Yasmin Paterson, Deborah Guest

**Affiliations:** 1 Centre for Preventive Medicine, Animal Health Trust, Newmarket, Suffolk, CB8 7UU UK; 2 Department of Veterinary Medicine, University of Cambridge, Cambridgeshire, CB3 OES UK

## Abstract

Interleukin 1β (IL-1β) is upregulated following tendon injury. Here we demonstrate that in adult and fetal tenocytes IL-1β increases the expression of matrix metalloproteinases, tenascin-C and Sox9 and decreases the expression of scleraxis and cartilage oligomeric matrix protein. When cultured in 3-dimensional collagen gels adult and fetal tenocytes exposed to IL-1β have reduced contraction ability and generate tendon-like constructs with a lower storage modulus. In contrast, equine embryonic stem cell (ESC) derived tenocytes exposed to IL-1β exhibit no changes in gene expression and generate identical tendon-like constructs. We propose that ESC-derived tenocytes do not respond to IL-1β due to their low expression of interleukin 1 (IL-1) receptor 1 and high expression of the decoy receptor IL-1 receptor 2 and IL-1 receptor antagonist protein (IL1Ra). This may make ESC-derived tenocytes an advantageous source of cells for tissue regeneration and allow the development of novel pharmaceutical interventions to protect endogenous cells from inflammation.

## Introduction

Tendon injuries are one of the most common orthopaedic injuries in human and equine athletes. They also occur in human non-athletes and are estimated to account for 30–50% of all musculoskeletal injuries^1^. The long recuperation periods required following a tendon injury can have a large financial impact. The structure and function of tendons are very similar in horses and humans and they share many of the same risk factors for tendon injuries such as age and training. Horses therefore provide a relevant large animal model for studying the human injury process and evaluating novel therapies^2^.

Adult tendon injuries in both species undergo poor natural regeneration, healing via the formation of scar tissue which is biomechanically inferior to healthy tendon and pre-disposes the individual to re-injury rates of up to 67% in horses^3^. In contrast, fetal tendon injuries have been reported to heal via regeneration in the absence of any scar tissue^4^. This is due to intrinsic properties of the fetal tendon itself, as injured fetal tendons transplanted into an adult environment continue to regenerate^5^. Furthermore, fetal tenocytes give better tissue repair than adult tenocytes suggesting regeneration is controlled at the cellular level^6^. Regenerative medicine methodologies to encourage the fetal-like regeneration of adult tendon tissue after an injury are therefore being investigated and biological products such as mesenchymal stem cells (MSCs)^7^ and platelet rich plasma (PRP)^8^ are already widely available for equine veterinary use.

We have previously derived equine embryonic stem cells (ESCs) from very early horse embryos 7 days after fertilisation^9,10^. ESCs have the potential to turn into derivatives of all three germ layers^11^. In contrast, fetal tenocytes from early development show some plasticity^12^, but at later stages of development only the small population of tendon stem cells retain some multipotent properties and can differentiate into cartilage, bone and fat^13,14^. ESCs can differentiate into tenocytes in response to transforming growth factor beta 3 (TGFβ3), 3D culture^15,16^ or *in vivo* implantation into horse tendon lesions^17^, in a process which is dependent on the transcription factor scleraxis (SCX)^18^. Furthermore, equine ESCs and their differentiated progeny do not stimulate the proliferation of allogeneic immune cells *in vitro*^19^. ESCs may therefore provide an unlimited, ready to use source of cells for allogeneic transplantation therapies. However, prior to their clinical use, it is critical to understand if they will be safe and effective when exposed to the injured tissue environment.

Historically, tendon injuries were believed to be devoid of inflammation. However, it is now understood that inflammation is present in both horse and human tendon injuries^20,21^ and a range of inflammatory cytokines have been found to be upregulated after an injury^22^. Of these, IL-1β is upregulated in injured equine tendons^23^ and has negative effects on gene expression in cultured tenocytes from other species; increasing the expression of inflammatory factors such as cyclooxygenase 2 (COX2), interleukin-6 (IL-6) and tumour necrosis factor alpha (TNFα), increasing the expression of matrix degrading enzymes such as matrix metalloproteinases (MMPs) and aggrecanases, and decreasing the expression of factors required for matrix production such as collagens, biglycan and decorin^24–26^. However, it is not clear if these changes in gene expression lead to any detrimental changes in tenocyte function.

Human and mouse ESCs and their differentiated progeny (including fibroblasts, endothelial cells, smooth muscle cells, cardiomyocytes, osteoblasts) do not appear to have an active inflammatory response mechanism^27,28^. While this may leave them more sensitive to infectious agents, it may provide them with a therapeutic advantage if inflammatory signals have negative effects on adult cells. In this study we hypothesised that the inflammatory cytokine IL-1β would impair adult tenocyte gene expression and collagen gel contraction, but that equine ESC-derived tenocytes would not respond to IL-1β and show no changes in gene expression or collagen gel contraction.

## Results

### IL-1β increases the expression of MMPs in adult and fetal but not ESC-tenocytes

**Figure 1:**
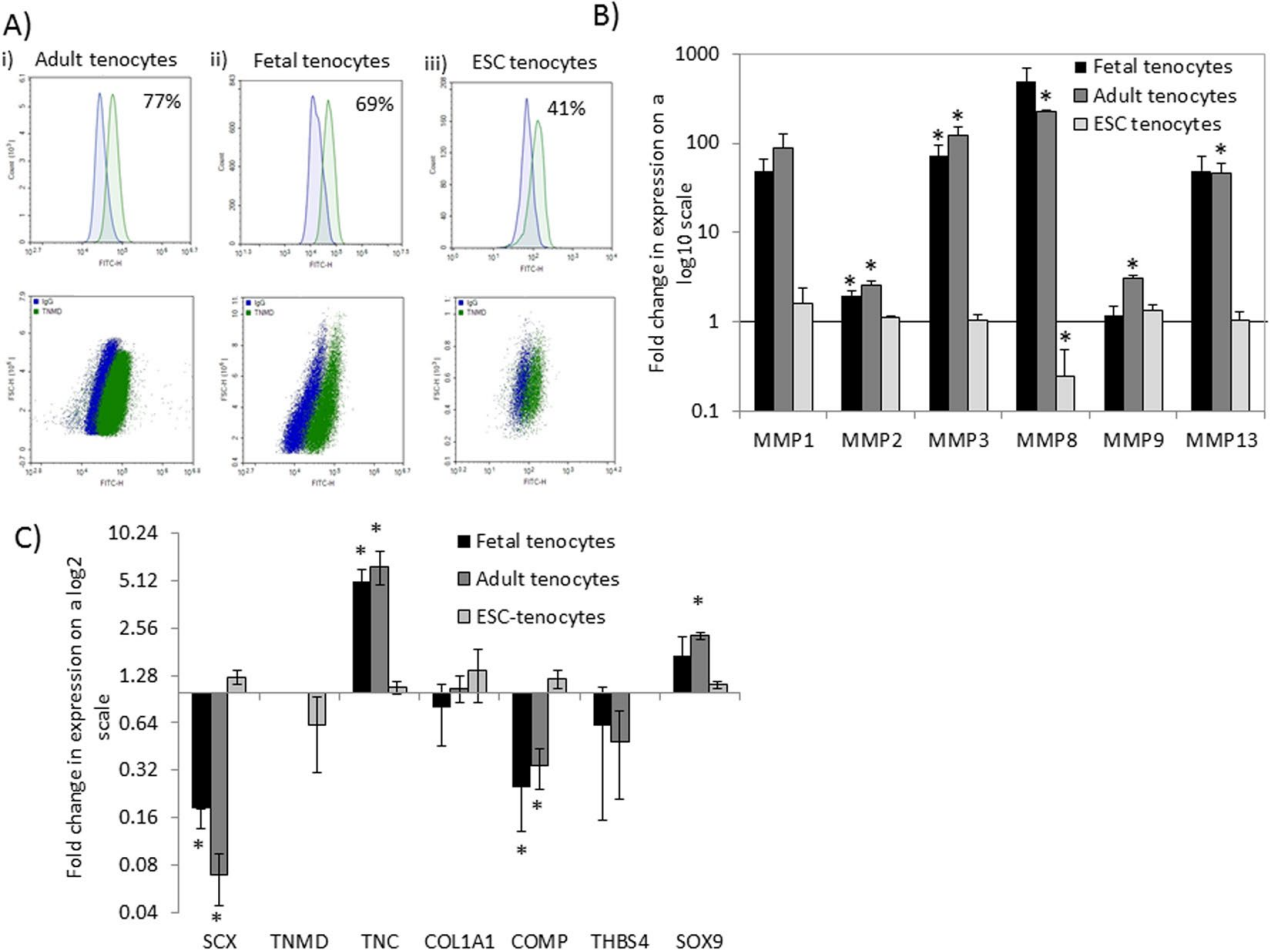
IL-1β exposure of adult, fetal and ESC-tenocytes results in different gene expression responses. (**A**) Representative flow cytometry histograms and dot plots of TNMD expression from three biological replicates of (i) adult, (ii) fetal and (iii) ESC-tenocytes cultured in 2D. Blue represents isotype control, green represents TNMD. (**B**,**C**) Fold change in gene expression in fetal, adult and ESC-tenocytes following IL-1β exposure for 72 h compared to control cells (fetal, adult or ESC-tenocytes not exposed to IL-1β) on a log scale. Error bars represent the s.e.m. of three independent biological replicates. *p < 0.05 using an unpaired Student’s t-test.

The characterisation of the ESC-derived tenocytes following 2D^15^ and 3D^16^ differentiation has been described previously. This demonstrated a clear change in morphology and the expression of tendon-associated genes and their corresponding proteins using quantitative polymerase chain reaction (qPCR) and immunocytochemistry. In this study we quantified the percentage of ESC-derived tenocytes that expressed a mature tendon cell marker, tenomodulin (TNMD) using flow cytometry analysis. This demonstrated that, after 2D *in vitro* differentiation, 41% of ESCs expressed TNMD. This is in comparison to 77% of adult tenocytes and 69% of fetal tenocytes (Fig. 1A).

After 72 h, IL1-β produced large increases in the expression of matrix metalloproteinases (MMP) 1, 3, 8 and 13 in adult and fetal tenocytes. These genes were consistently upregulated to a high degree in all replicates, however, due to the variation in the fold increase between biological replicates, not all changes were significant. Smaller, but still significant, increases in MMP2 are observed in both fetal and adult tenocytes. In adult tenocytes there is also a small but significant increase in MMP9 (Fig. 1B). In contrast, the only significant change in MMP gene expression in ESC-tenocytes is a small (3 fold) reduction in MMP8.

### IL-1β changes tendon-associated gene expression in adult and fetal but not ESC-tenocytes

After 72 h of IL-1β treatment adult and fetal tenocytes significantly decrease the expression of scleraxis (*SCX*) and cartilage oligomeric matrix protein (*COMP*) and significantly increase the expression of Tenascin-C (*TNC*). In adult tenocytes there was also a significant increase in expression of the cartilage progenitor marker, *SOX9* (Fig. 1C). In contrast, there were no significant changes in the expression of the tendon-associated genes or *SOX9* in ESC-tenocytes (Fig. 1C).

### IL-1β reduces collagen gel contraction in 3D cultures by adult and fetal but not ESC-tenocytes

**Figure 2:**
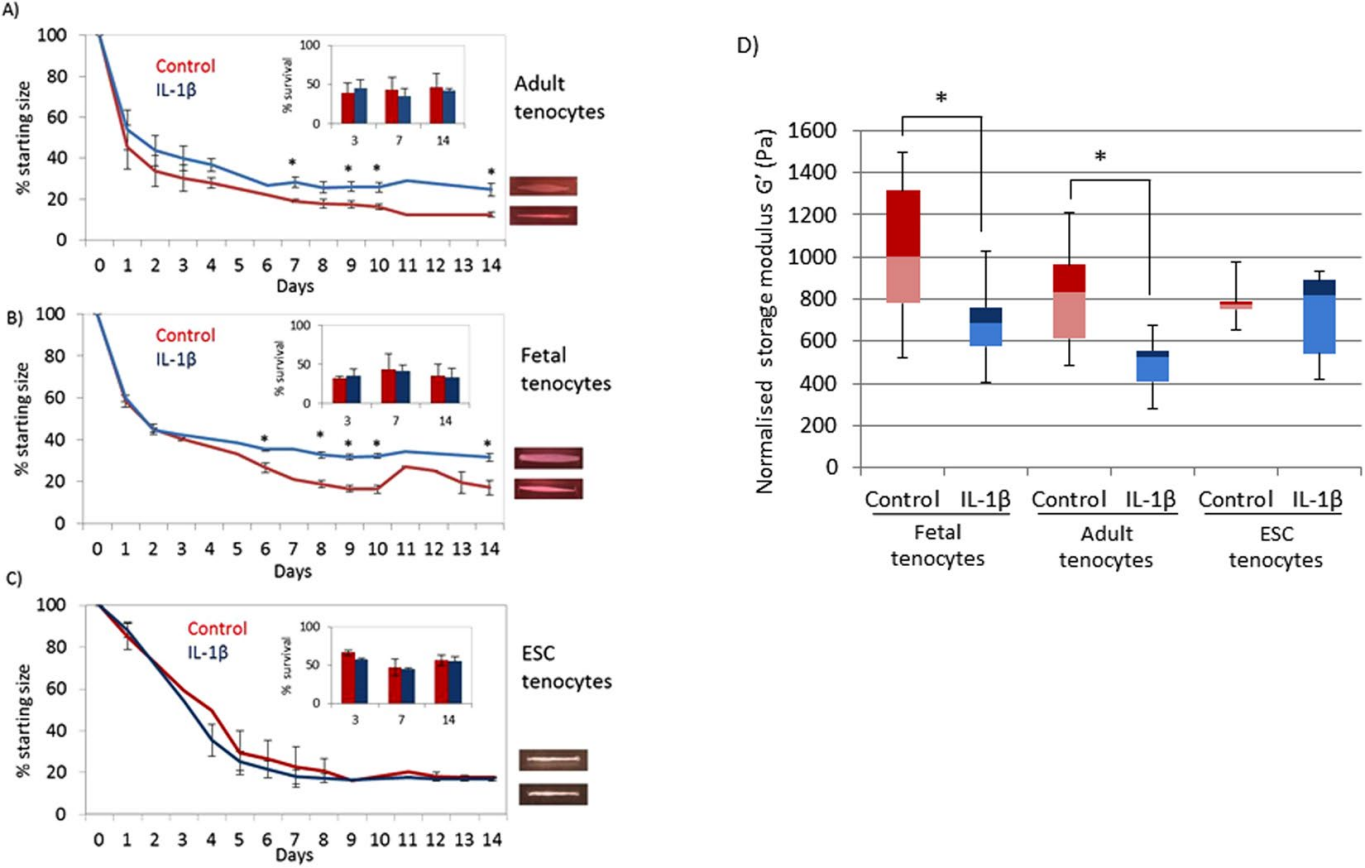
IL-1β reduces collagen gel contraction by adult and fetal but not ESC-tenocytes. Collagen gels containing (**A**) adult and (**B**) fetal tenocytes remain significantly larger in the presence of IL-1β (blue) than adult and fetal tenocytes cultured in the absence of IL-1β (red) but collagen gel contraction by ESC-tenocytes (**C**) is not affected in IL-1β treated ESC-tenocytes (blue) compared to non-treated control ESC-tenocytes (red). (**D**) The storage modulus of tendon-like constructs generated by adult and fetal, but not ESC-tenocytes, is significantly lower in the presence of IL-1β (blue bars) than in control adult and fetal cells cultured in the absence of IL-1β (red bars). Storage modulus is normalised to the size of the starting material. Error bars represent the s.e.m. of three independent biological replicates. *p < 0.05 using an unpaired Student’s t-test.

When cultured in 3D collagen gels the degree of gel contraction by adult and fetal tenocytes is significantly impaired in the presence of IL-1β. This is not due to differences in cell survival as no significant differences in cell numbers were observed (Fig. 2A,B). In contrast, 3D cultures containing ESC-tenocytes exhibit no significant differences in collagen gel contraction (Fig. 2C). There are no significant differences in the final degree of contraction reached by any of the cells in control culture conditions lacking IL-1β, however after 1 day of culture the adult and fetal collagen gels without IL-1β have undergone significantly more contraction than the ESC seeded collagen gels without IL-1β.

Dynamic shear analysis (DSA) demonstrated that the resulting adult and fetal tendon-like constructs exposed to IL-1β had a significantly lower storage modulus (G’) than the control constructs but G’ was unaffected by exposure to IL-1β in ESC-tenocyte constructs (Fig. 2D).

### IL1 receptor antagonist (IL1Ra) can restore collagen gel contraction by adult and fetal tenocytes in 3D cultures

**Figure 3:**
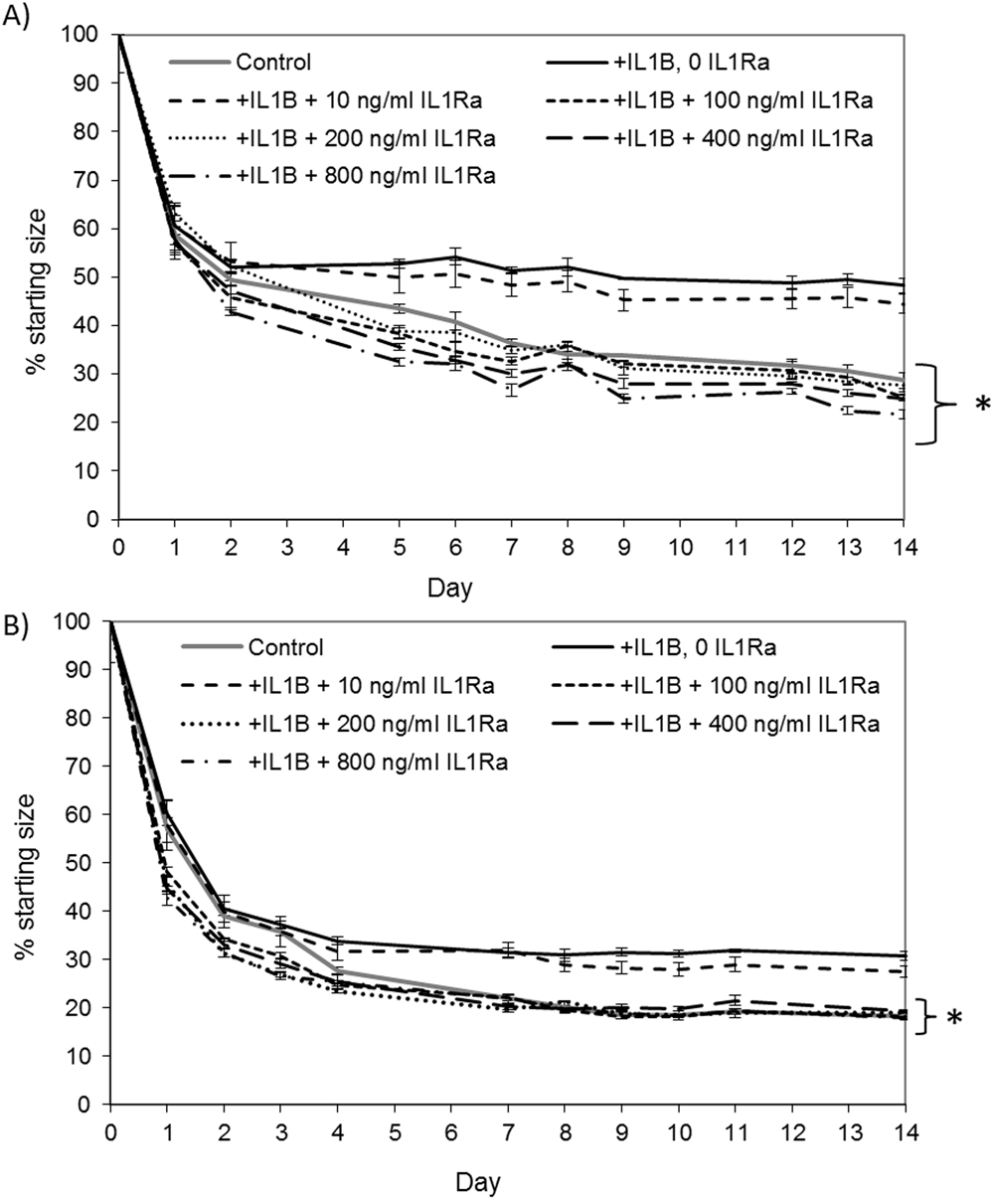
IL1 receptor antagonist (IL1Ra) can protect adult and fetal tenocytes from 17 ng/ml IL-1β when used at 100 ng/ml or more. Three biological replicates were carried out for each cell type and a representative example of collagen gel contraction is shown from one line of (**A**) adult and (**B**) fetal tenocytes. Error bars represent the s.e.m. of three technical replicates. *p < 0.05 indicates the final degree of contraction is significantly different to the presence of 17 ng/ml IL-1β and no IL1Ra using an unpaired Student’s T-test.

The addition of IL1Ra can block the effect of IL-1β on collagen gel contraction by adult and fetal tenocytes, but only when used at 6 times or more the amount of IL-1β (Fig. 3). The use of IL1Ra on its own has no significant effects on matrix contraction or cell survival at any of the concentrations tested (Supplementary Fig. 1).

### ESC-tenocytes express different levels of IL-1β signalling components compared to fetal and adult tenocytes and do not translocate NFκB in response to IL-1β

**Figure 4:**
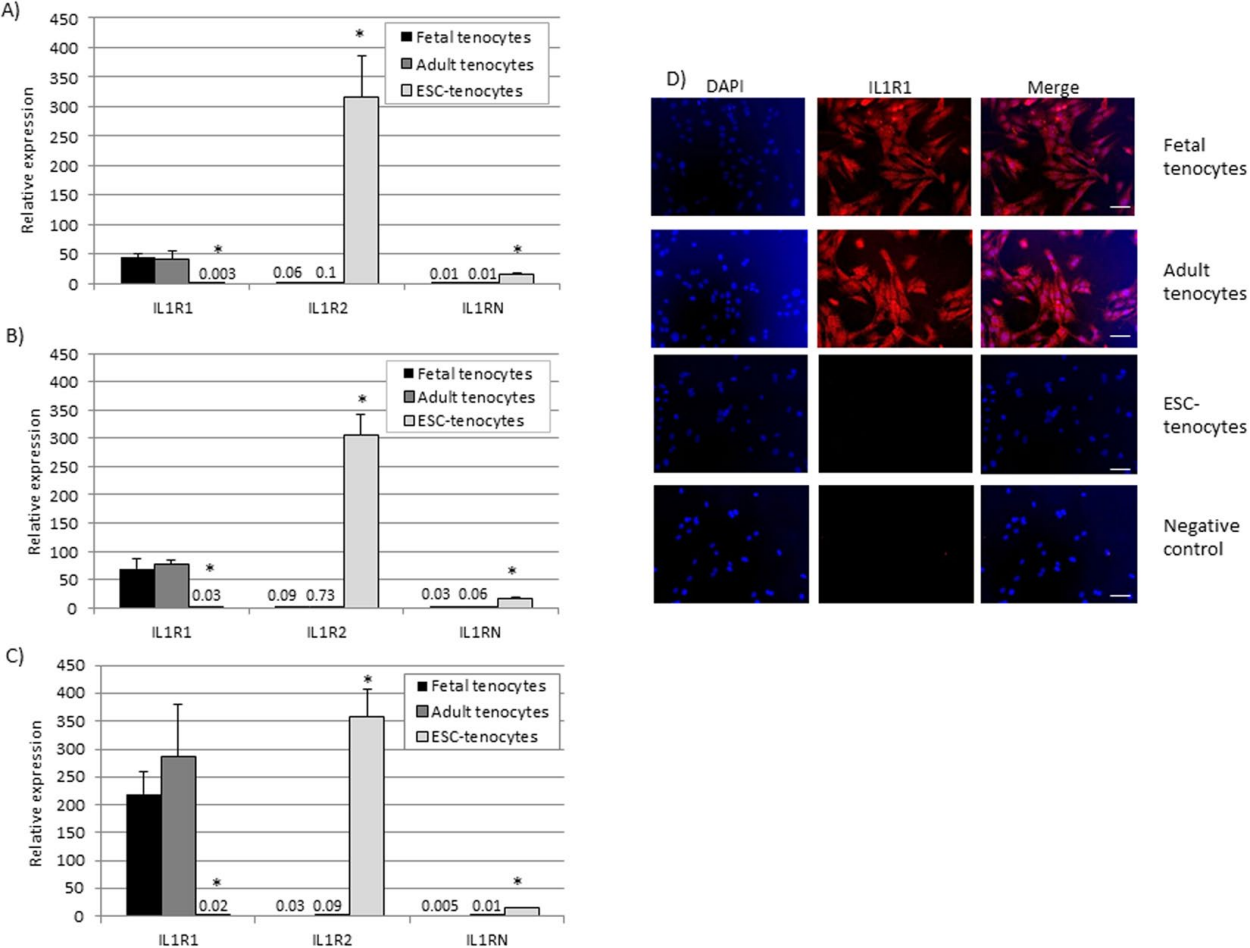
Expression of IL-1β receptors. Adult and fetal tenocytes express significantly more *IL1R1* and less *IL1R2* and *IL1RN* than ESC-tenocytes when cultured in 2D in (**A**) the absence of IL-1β, (**B**) the presence of IL-1β and (**C**) 3D culture. The relative expression compared to the housekeeping gene (18 s) is shown. Error bars represent the s.e.m. of three independent biological replicates. *p < 0.05 using a single factor ANOVA with *post hoc* Tukey test. (**D**) Immunocytochemistry demonstrates the presence of IL1R1 protein (red) in the fetal and adult tenocytes but not in the ESC-tenocytes. DAPI staining of the nuclei is shown in blue. Scale bar = 20 µm.

ESC-tenocytes cultured in 2D in the absence of IL-1β express significantly less of the IL-1β signalling receptor *IL1R1* and significantly more of the decoy receptor *IL1R2* and the gene encoding endogenous IL1Ra, *IL1RN* than adult and fetal tenocytes (Fig. 4A). 72 h of exposure to IL-1β does not significantly affect the expression levels of these genes (Fig. 4B). In contrast, 3D culture (in the absence of IL-1β) results in a significant increase in *IL1R1* expression by fetal and adult tenocytes, but not ESC-tenocytes (Fig. 4C). Correspondingly, immunocytochemistry on cells cultured in 2D in the absence of IL-1β demonstrates the presence of IL1R1 protein in fetal and adult tenocytes but not ESC-tenocytes (Fig. 4D).

**Figure 5:**
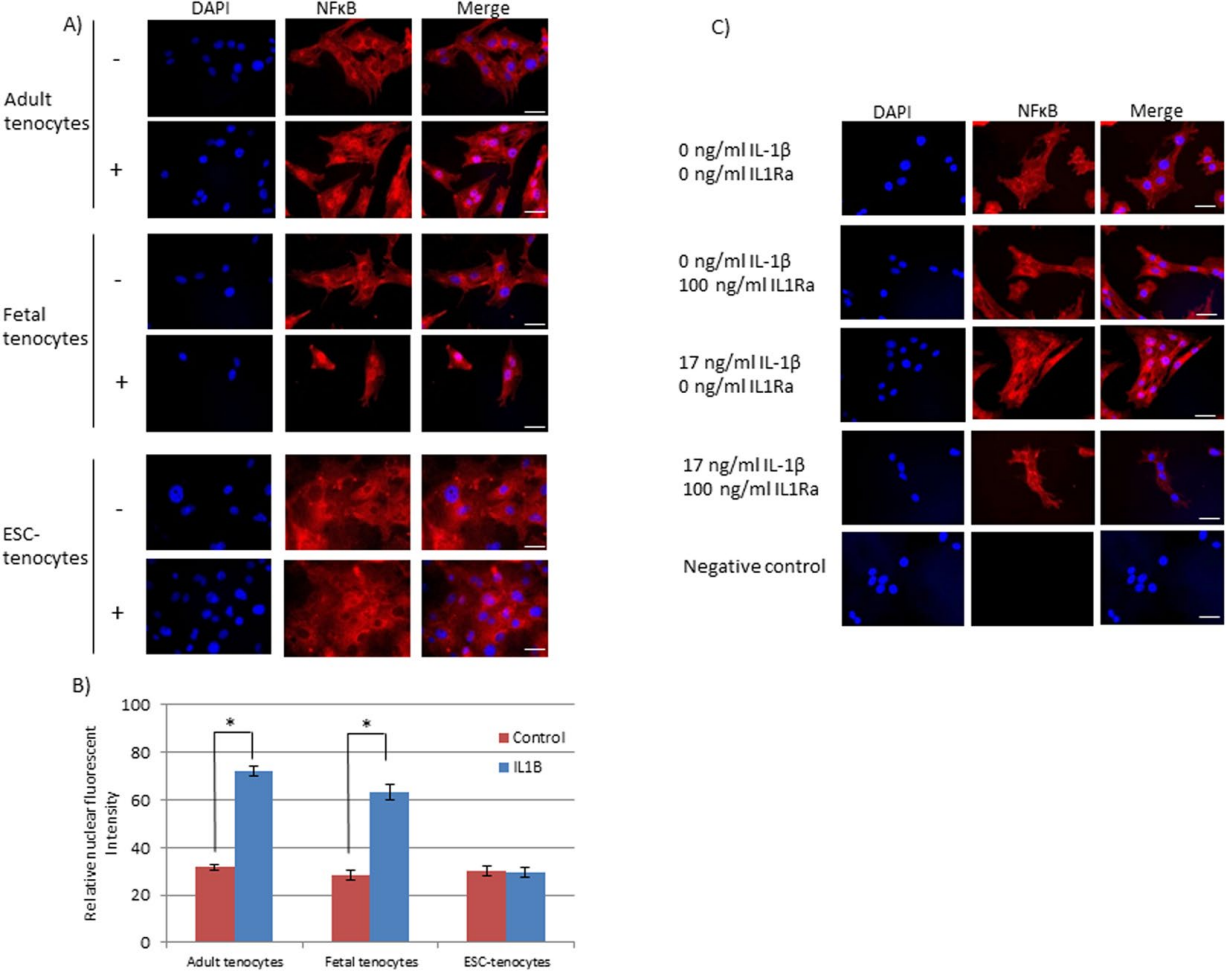
The localisation of IL-1β signalling molecule NFκB. (**A**) NFκB is translocated to the nucleus of (i) adult and (ii) fetal tenocytes following 20 minutes of exposure to IL-1β but remains in the cytoplasm in ESC-derived tenocytes. Scale bar = 20 µm. (**B**) Quantification of the relative nuclear fluorescent intensity demonstrates that it is significantly increased in adult and fetal tenocytes following IL-1β exposure compared to untreated cells. Error bars represent the s.e.m. of three measurements from each of three biological replicates. *p < 0.05 using an unpaired Student’s T-test. (**C**) NFκB translocation into the nucleus of adult tenocytes in response to 20 minutes of exposure to 17 ng/ml IL-1β can be blocked by the simultaneous addition of 100 ng/ml IL1Ra. Scale bar = 20 µm.

In adult and fetal tenocytes 20 minutes of IL-1β treatment results in the rapid translocation of nuclear factor kappa-light-chain-enhancer of activated B cells (NFκB) from the cytoplasm into the nucleus. In contrast, in ESC-tenocytes, NFκB retains its cytoplasmic localisation (Fig. 5A,B). The translocation of NFκB in response to 17 ng/ml IL-1β can be blocked in adult tendon cells by the co-application of 100 ng/ml IL1Ra (Fig. 5C).

## Discussion

IL-1β is upregulated in tendon injuries and may have negative consequences for endogenous tendon cells and those applied during a cell based therapy. We have demonstrated that IL-1β affects gene expression by adult and fetal tenocytes and their functional ability to contract a collagen gel and generate tendon-like constructs.

IL-1β upregulates MMP1, 3 and 13 gene expression in adult human^24^, mouse^25^ and rabbit^26^ tenocytes. We have confirmed that both adult and fetal equine tenocytes upregulate MMP1, 2, 3, 8, 9 and 13. However, whether or not this is reflected in increased MMP activity is still to be determined.

Fetal and adult equine tenocytes also show changes in other genes following IL-1β exposure, exhibiting a decrease in *SCX* and *COMP* and an increase in *TNC* and *SOX9*. SCX is a transcription factor that regulates tendon genes and is required for normal tendon development^29–32^ and adult tendon repair in mice^33^. Therefore a reduction in its expression is likely to result in changes in the expression of its downstream genes and be detrimental to tendon healing post injury. COMP is a proteoglycan that promotes cell attachment^34^, binds to collagen to assist extra-cellular matrix assembly^35^ and may help the tendon to resist compression^36^. Decreased COMP expression during tendon healing may therefore delay the assembly of the new ECM and contribute to the reduced strength of the repair tissue. *TNC* encodes Tenascin-C, a glycoprotein that can drive persistent fibrosis^37,38^. In tendon tissue, TNC has been found to be associated with degeneration by driving fibrocartilage formation^39^. SOX9 is a key driver of cartilage differentiation^40^ and its upregulation in a tendon healing model has been shown to lead to cartilage-like tissues being formed and ectopic ossification^33^. Therefore, together the observed changes in gene expression in response to IL-1β are likely to have a negative impact on the cells and their ability to regenerate a healthy tendon matrix. No significant changes were observed in the expression of *TNMD*, *COL1A1* or *THBS4* in either the fetal or adult tenocytes exposed to IL-1β compared to control, untreated cells. Interestingly, no *TNMD* mRNA was detected either with or without IL-1β in the fetal and adult tenocytes. This may be due to the passage number as *TNMD* mRNA decreases in cultured tenocytes with passage^29^. The detection of TNMD protein in this study in the absence of mRNA has been demonstrated previously^15,18^ and suggests that TNMD mRNA and protein have different stabilities^41^.

We further show that IL-1β significantly inhibits the rate of collagen gel contraction by adult and fetal tenocytes in 3D cultures. Collagen gel contraction is widely used as a measure of cell-mediated matrix reorganisation^42–45^ and is directly affected by MMP expression in fibroblasts^46^ and smooth muscle cells where it has a dose dependent effect on collagen gel contraction with low levels of MMP9 giving increased contraction, but high levels of MMP9 resulting in lower contraction^47^. The level of collagen degradation, in response to MMP activity, has also been shown to be directly linked to the degree of collagen gel contraction in lung fibroblasts^48^. Our data suggest that the increased MMP expression in response to IL-1β in equine adult and fetal tenocytes results in a faster rate of collagen degradation than production. However, MMP activity and collagen levels in the gels must be directly measured to determine if this is the case. To determine the effects of IL-1β on matrix production, it may be beneficial to use a fibrin gel system^49,50^ to allow collagen production to be determined^51^. The reduction in collagen gel contraction by adult and fetal tenocytes cultured in the presence of IL-1β can be rescued through the addition of IL-1Ra, but only when used at six times or more the concentration of IL-β.

IL-1β treatment of fetal and adult tenocytes also reduces the storage modulus of the resulting tendon-like constructs. This was not due to any changes in overall cell survival. Tendon cell survival has been shown to be decreased in response to IL-1β in some studies^25^, but not others^52^, which may reflect whether or not the cells were serum starved during the IL-1β exposure. Direct measurement of cell proliferation and cell death in the collagen gels in the presence and absence of IL-1β may be beneficial. Human tenocytes exposed to IL-1β have also been shown to produce tendon-like constructs that have a reduced modulus^53^. This corresponded to an increased elasticity which the authors proposed may have beneficial effects to prevent tendon rupture. However, they also reported that the ultimate tensile strength (UTS) of the tendon-like constructs was reduced. We did not measure UTS in our study. DSA was used to measure the storage modulus of the tendon-like constructs as traditional tensile testing failed on a large proportion of tendon-like constructs, due to their small size and difficult to grip nature. The implementation of DSA for calculating the storage modulus of such tissue samples has been reported previously^54,55^. As traditional tensile testing could not be performed we were unable to measure the UTS of the tendon-like constructs. However, in tendon tissue the UTS and elastics modulus correlate very strongly^56^ and therefore we expect that the reduced modulus of the tendon-like constructs in response to IL-1β would correlate with a reduced UTS. Functionally, a repaired tendon that had a reduced modulus would take longer to transmit a force and the reduced UTS would lead to a higher chance of rupture when exposed to high forces. Therefore, together the reduced collagen gel contraction capacity and altered gene expression in adult and fetal tenocytes would suggest that IL-1β has negative effects on the regenerative capacity of tenocytes from both stages of development.

Fetal tenocytes were used in this study as fetal tendon, in contrast to adult tendon, undergoes regeneration following injury rather than scar tissue mediated repair^4^. Fetal tendon injuries have lower levels of inflammation than adult tendon injuries^5^. However, regeneration occurs even when fetal tendon is transplanted into an adult environment, suggesting that it is an intrinsic property of the tendon itself and is not inhibited by the inflammatory adult environment^5^. Furthermore, fetal tenocytes transplanted into an adult tendon injury produce better tissue regeneration than adult tenocytes^6^. In these transplantion studies, fetal tendon tissue and cells were isolated from fetuses around 50–55% of the way through gestation whereas in this study the equine fetal tenocytes were from fetuses around 80–90% of the way through gestation (322–387 days in a horse). Fetal innate immunity is already developed at the time of birth in horses^57^ and our data demonstrates that fetal tenocytes taken towards the end of gestation are as responsive to IL-1β signalling as the adult tenocytes. Further work is required to determine if tenocytes isolated from younger fetuses exhibit a different response to IL-1β.

In contrast to fetal and adult tenocytes, equine ESC-tenocytes exhibited no changes in gene expression or collagen gel contraction following IL-1β exposure, with the exception of a small (3-fold) but significant reduction in MMP8 expression. MMP8 is a collagenase and is therefore likely to be involved in matrix remodelling following an injury. However, a single nucleotide polymorphism in the promoter region of human MMP8 that increases gene expression, is associated with a higher risk of tendon injury^58^. Therefore a reduced level of MMP8 expression may be beneficial.

We have previously demonstrated that equine ESCs can differentiate into tenocytes in 2D culture in response to TGF-β3^15^ and in 3D culture in the presence and absence of TGF-β3^16^. We demonstrated that following 2D differentiation, the ESCs exhibit a change in morphology, a rapid (24 h) induction of the tendon progenitor marker *SCX*, and after 14 days of differentiation they express tendon-associated genes and proteins including SCX, TNC, COL1A1 and COMP. However, we had not previously quantified the proportion of tendon cells produced. Here we demonstrate that when cultured in 2D a homogenous population of tenocytes is not produced; around 40% of the ESC-derived cells express the tendon-associated marker TNMD in contrast to around 70% of fetal and adult tenocytes. As TNMD is a late tendon marker, future work to determine the proportion of cells expressing an early tendon marker, such as SCX, may enable further characterisation of the heterogenous population produced. It is likely that this population contains a mixture of early tendon cells (TNMD negative), late tendon cells (TNMD positive) and non-tendon cells (TNMD negative). We therefore cannot rule out the possibility that alterations in MMP gene expression did occur in the mature tendon cells but that they were not detected due to the heterogeneous population. However, we also did not observe any changes in tendon-associated genes following IL-1β exposure and these genes are only likely to be expressed in tendon cells. Furthermore, we found no change in the collagen gel contraction capacity of ESC-tenocytes in 3D culture upon exposure to IL-1β. In the absence of IL-1β, ESCs can contract the collagen gel to the same degree as adult and fetal cells after 14 days of culture, however, ESCs display a significantly slower rate of contraction after 1 day of culture. This is likely because the collagen gels are seeded with undifferentiated ESCs and there is a delay in collagen contraction while the cells undergo tenocyte differentiation. ESCs differentiate more efficiently in 3D than in 2D culture and immunohistochemical studies have demonstrated that the majority of cells express the late tendon cell maker TNMD^16^. Unfortunately we were not able to quantify TNMD positive cells following 3D differentiation as digestion of the constructs to release the cells resulted in the destruction of the antibody epitope. With 40% of the population expressing the later tendon marker TNMD, we would also have expected to see NFκB translocation in almost half the cells. However, we did not observe this in any of the ESC-derived cells treated with IL1-β. This suggests that the progeny of ESCs do not activate NFκB signalling in response to IL-1β whether they are in early or late stages of *in vitro* tendon differentiation. Isolation of specific populations of cells using fluorescent or magnetic activated cell sorting would allow this to be determined in the future.

The lack of an inflammatory response of ESCs has been shown in a variety of cell types produced from mouse ESCs^59,60^ but this is the first report in equine ESCs and for tenocyte-lineage differentiation. Mouse ESCs and their differentiated derivatives (produced by non-directed differentiation) are protected from the effects of inflammatory cytokines, including IL-1β, due to a failure to activate NFκB^28^. In this study we investigated the mechanisms by which ESCs are protected from IL-1β by examining the expression of the IL-1β signalling receptors. The *IL1R1* gene encodes the type I IL-1 receptor (IL-1R1) that, along with the IL-1 receptor accessory protein (IL-1RAP), creates a transmembrane signalling complex to initiate the IL-1β signalling cascade. IL-1 receptor antagonist (encoded by the *IL1RN* gene) can bind to IL-1R1 to block this signalling pathway. The *IL1R2* gene encodes the type 2 IL-1 receptor (IL-1R2). This receptor acts as a decoy as its affinity for IL-1β is similar to that of IL-1R1 but it lacks the intracellular signalling domain^61^. In this study we found that ESC-tenocytes consistently expressed significantly lower levels of *IL1R1* and higher levels of *IL1R2* and *IL1RN* than adult and fetal tenocytes. At the protein level, IL1R1 was detected in the fetal and adult tenocytes but was undetectable in the ESC-tenocytes, reflecting the low level of gene expression. Unfortunately no antibody to IL1R2 was found that cross-reacted with the horse receptor and so the gene expression results for IL1R2 are yet to be confirmed at the protein level. Although some cell types upregulate the expression of *IL1R1*, *IL1R2* and *IL1RN* following exposure to IL-1β^62^, this had no effect in any of the tenocyte types. In contrast, both adult and fetal tenocytes (but not ESC-tenocytes) significantly upregulated *IL1R1* expression in 3D culture compared to 2D culture. To our knowledge, this is the first time reduced *IL1R1* and increased *IL1R2* and *IL1RN* expression has been reported in any ESC-derived cell type from any species and it remains to be determined if this expression pattern is maintained in other cell lineages derived from ESCs.

We further demonstrated that within 20 minutes of IL-1β exposure, adult and fetal tenocytes translocate NFκB from the cytoplasm to the nucleus where it can activate gene transcription and that this translocation can be blocked using a 6 fold higher concentration of IL1Ra. This would suggest that IL1Ra may inhibit changes in gene expression as well as in collagen contraction. However this must be confirmed in future work. In contrast to the fetal and adult tenocytes, NFκB is not translocated in ESC-derived tenocytes, similar to reports in mouse^28^ and human ESCs^63^. In addition to the activation of NFκB, the binding of IL-1β to its receptor can also activate c-Jun N-terminal protein kinase (JNK) and p38 mitogen-activated protein kinase pathways (p38 MAPK)^64^. They are also translocated to the nucleus where they can initiate activation of gene transcription through complex formation with activator protein 1 (AP1). In this study we only determined the effect of IL-1β signalling on NFκB activation, since this has previously been found to be inhibited in mouse ESC-derivatives^28^. Future work to determine if JNK and p38 MAPK are also involved in IL-1β signalling in equine tenocytes is required.

The lack of an inflammatory response by ESCs may be because any negative effects on proliferation and differentiation could have a severe impact during early development^27,28^. This may provide an advantage to ESC-derived cell based therapies where inflammation is likely to be present in the injured tissue, although whether or not this may have a longer term impact on the transplanted cells if they cannot respond to infection^65^ is still to be determined. It is also unclear if longer periods of *in vivo* differentiation would result in the cells developing the ability to respond to inflammation over time. Other hurdles to the clinical translation of ESC-derived cells also remain, such as ensuring their commitment to the tendon lineage to prevent uncontrolled proliferation and differentiation. However, our results also open up the possibility of using ESC-derived tenocytes as an *in vitro* tool to enable the development of novel pharmaceuticals to protect the endogenous adult cells against inflammatory cytokines without using cell based products.

In horses, autologous conditioned serum (ACS) and IRAP (Interleukin Receptor Antagonist Protein) have been marketed as a source of IL1Ra for the treatment of joint inflammation. However, they contain variable levels of the candidate active molecule IL1Ra^66^ and there are limited studies on their effectiveness in horse tendon injuries^67,68^. In humans, Anakinra, a recombinant IL1Ra, is used to treat rheumatoid arthritis. However, it has a short *in vivo* half-life and must be injected daily^69^ and at high concentrations (100–1000 fold excess relative to IL-1β) to work effectively^70^. Similarly, we have demonstrated that *in vitro*, IL1Ra does not protect the collagen gel contraction ability of adult or fetal tenocytes unless it is used six-fold higher than the concentration of IL-1β. This is because the IL1R1 receptor has very similar binding affinities for both IL1Ra and IL-1β^71^. Other more effective interventions are therefore required and further interrogation of the mechanisms by which *in vitro* differentiated ESCs are protected from inflammation may enable new strategies to be developed.

## Conclusions

Here we demonstrate that equine ESC-tenocytes are protected from the negative consequences of IL-1β signalling through their unique expression of IL-1 receptors. Future work to determine if this results in better *in vivo* tendon regeneration can now be performed in a relevant large animal model. Furthermore, understanding the mechanisms by which ESC-derived tenocytes are protected may lead to the development of new pharmaceuticals to protect endogenous tendon cells from inflammation and enable better tissue regeneration.

## Methods

### 2D cell culture

Tenocytes were isolated post-mortem from three adult Thoroughbred horses and three fetuses at 319, 320 and 321 days of gestation which had undergone spontaneous abortion. Tenocytes were isolated and cultured as described previously^15^. Tendon tissue was digested in 1 mg/ml type I collagenase (Sigma-Aldrich, Dorset, UK) overnight and the cells were cultured in Dulbecco’s modified eagle medium (DMEM) high glucose, 10% fetal bovine serum, 2 mM L-glutamine, 100 U/ml penicillin, 100 µg/ml streptomycin (all from Invitrogen, Renfrewshire, UK). Equine tenocytes were passaged using 0.25% trypsin-EDTA (Sigma-Aldrich) every 3–4 days just prior to reaching confluency.

Characterised equine ESCs^9,15–18^, from three different embryos, were cultured and differentiated in 2D as described previously^15^. To maintain their undifferentiated state, ESCs were cultured on mitotically inactivated mouse embryonic fibroblasts at 37 °C, 5% CO_2_ in ESC medium (DMEM/F12 containing 15% fetal bovine serum, 2 mM L-glutamine, 1% non-essential amino acids, 1 mM sodium pyruvate, 0.1 mM 2-mercaptoethanol (all from Invitrogen) and 1000 units/ml leukaemia inhibitory factor (LIF) (Sigma-Aldrich). ESCs were passaged mechanically every 5–7 days in the presence of 2 μM Thiazovivin (StemGent, Cambridge, USA). To differentiate the ESCs in 2D culture, they were plated without feeders in ESC media lacking LIF and in the presence of 20 ng/ml TGF-β3 (Peprotech, London, UK) and cultured for 14 days.

Tenocytes from adult, fetal and ESCs were exposed to 0 ng/ml (control) and 17 ng/ml human recombinant IL-1β (Peprotech) for 72 h prior to the harvest of the cells for RNA. Adult and fetal tenocytes were used between passages 4 and 11 for all experiments, ESCs were used between passages 17 and 20. All experiments were performed using fetal tenocytes, adult tenocytes and ESCs derived from three different horses (i.e. three biological replicates for each cell type).

### 3D cell culture

3D cell culture in collagen gels was performed for 14 days as described previously^16,18,72^. 0.2 mm diameter minutien pins were embedded in silicone coated 6-well plates (Sylgard 184 Silicone elastomer, Dow Corning, USA) in pairs 15 mm apart. 4 × 10^5^ cells/ml were suspended in a chilled mixture of 2 parts medium, 8 parts PureCol (Bovine collagen type I, Advanced Biomatrix, USA) (with the pH adjusted to 7.2 to 7.6). 200 µl of collagen-cell suspension was used per construct between each pair of minutien pins. The 6-well plates were sealed with parafilm and kept at 37.5 °C for 60–90 minutes to allow the constructs to set prior to the addition of medium.

Contraction analyses were performed using ImageJ software (National Institutes of Health) and are displayed as a percentage of the Day 0 value. Cell survival was measured by digesting the constructs in 1 mg/ml type I collagenase (Sigma-Aldrich) for 1–2 hours at 37.5 °C and results are displayed as a percentage of the seeded cell number on Day 0. Gel contraction and cell survival were performed on three independent biological replicates with each replicate containing 1 to 9 tendon-like constructs. A Student’s t-test was used to determine statistically significant differences between treated cells compared to untreated control cells. IL-1β was used at a concentration of 0 ng/ml (control untreated cells) and 17 ng/ml (IL-1β treated cells) and was replaced every 3–4 days. Recombinant human IL1Ra (Sigma-Aldrich) was used at concentrations from 0–800 ng/ml and was also replaced every 3–4 days.

### Flow cytometry

Flow cytometry was performed on three biological replicates each of adult, fetal and ESC-derived cells. Cells were separated using 30 μm nylon filters (Miltenyi Biotec, Surrey, UK) and fixed in 3% paraformaldehyde (Sigma-Aldrich) for 20 minutes prior to washing. Blocking was carried out using 2.5% normal horse serum (Vector Laboratories, Peterborough, UK) for 20 minutes. 1 × 10^6^ cells were then incubated with either 2 μg/ml rabbit anti-TNMD (Santa Cruz, Heidelberg, Germany, SC-98875) or 2 μg/ml rabbit IgG (Vector Laboratories) for 45 min at 4 °C. Following washing, the cells were incubated with goat anti-rabbit FITC (1:100 dilution, Sigma, F1262) for 45 minutes at 4 °C. A maximum of 125,000 cell events were acquired on a NovoCyte (ACEA Biosciences, San Diego, USA) using NovoExpress software to analyse the samples as follows. Events were gated to exclude dead cells by forward versus side scatter height (R1). R1 events were gated to remove doublets (R2) by forward scatter height versus forward scatter area. 2% of events in the R2 isotype control and everything to the right were included in a new gate (R3) by FITC height versus forward scatter height. The overlayed TNMD incubated events gated within R3 were considered positive.

### Dynamic shear analysis

Dynamic shear analysis (DSA) was performed on three biological repeats, each in triplicate, as described previously^54^ using an AR 2000 rheometer (TA Instruments, New Castle, USA). Entire tendon-like constructs (after 14 days of culture in either the absence of IL-1β (control group) or the presence of 17 ng/ml IL-1β (IL-1β group)) were loaded in a coiled shape, and compressed to a minimum force of 0.02 N between two parallel plates. During testing samples were kept in PBS at 37 °C to prevent water loss whilst an oscillatory frequency sweep was performed (from 1 to 0.1 Hz at 1% strain). Average measures of G’ over this frequency range were compared. Sample area was determined by measurement of a post-test photograph in ImageJ. Data was normalised to reduce sample size as a variable. Unpaired Student’s t-tests were used to compare sample means.

### RNA extraction, cDNA synthesis and qPCR

RNA was extracted using Tri-reagent (Sigma), purified using the RNeasy mini kit (Qiagen, Manchester, UK) and treated with Ambion DNA-free (Life Technologies, Paisley, UK). cDNA was made from 1 μg of RNA using the sensiFAST cDNA synthesis kit (Bioline, London, UK). Primers were designed using primer3 (http://primer3.ut.ee/) and mfold (http://unafold.rna.albany.edu/?q = mfold) programs to obtain amplicons with a melting temperature (Tm) of 58 °C–62 °C, devoid a secondary structure at Tm 60 °C and with an amplicon size of 50–150 bp. Primer (Sigma-Aldrich) sequences can be found in Table 1. 2 μl aliquots of cDNA were used in qPCR using SYBR Green containing supermix (Bioline) on the Biorad C1000 Touch Thermal Cycler (Biorad, Hertfordshire, UK), and performed in duplicate. PCR cycle parameters were 95 °C for 10 min, followed by 40 cycles of 95 °C for 15 seconds, 60 °C for 15 seconds and 72 °C for 15 seconds. At the end of the program a melt curve was produced by taking readings every 1 °C from 65 °C to 95 °C. 18s rRNA levels did not change between treatments (data not shown) and were used to normalise gene expression using the 2^−ΔΔCt^ method^73^. When fold changes in gene expression are presented the relative expression of the IL-1β treated cells is compared to the control, untreated cells of the same type (adult, fetal or ESC-tenocytes). An unpaired student’s t-test or single factor ANOVA with *post hoc* Tukey test were used to determine significant differences in the mean gene expression using three biological replicates.

**Table 1:** Primer sequences used in quantitative PCR.

Gene	Forward primer	Reverse primer
18S rRNA	CCCAGTGAGAATGCCCTCTA	TGGCTGAGCAAGGTGTTATG
MMP1	CTTTGATGGACCTGGAGGAA	GAATGGCCAAATTCATGAGC
MMP2	CAGGAGGAGAAGGCTGTGTT	AGGGTGCTGGCTGAGTAGAC
MMP3	TGGACCTGGAAAAGTTTTGG	GACCAAGTTCATGAGCAGCA
MMP8	TTTGATGGACCCAATGGAAT	TTCATGGGCAGCAACAATAA
MMP9	GAGATCGGGAATCATCTCCA	CCAAGAGTCGCCAGTACCTC
MMP13	GCCACTTTGTGCTTCCTGAT	CGCATTTGTCTGGTGTTTTG
SCX	CCCAAACAGATCTGCACCTT	ATCCGCCTCTAACTCCGAAT
TNMD	GTCCCTCAAGTGAAGGTGGA	CCTCGACGGCAGTAAATACAA
TNC	AACCCGTCCAAAGAGACCTT	GCGTGGGATGGAAGTATCAT
COL1A1	TGCGAAGACACCAAGAACTG	GACTCCTGTGGTTTGGTCGT
COMP	AGAACATCATCTGGGCCAAC	CGCTGGATCTCGTAGTCCTC
THBS4	GGGAAATGGGGTTACCTGTT	CGGGTAGCAGGGATGATATT
SOX9	GCTCTGGAGACTTCTGAACGA	GTAATCCGGGTGGTCCTTCT
IL1R1	GAAGCGCATAAAGGGCACTA	CGTGGGCCTGATTTCATCTA
IL1R2	TCTGGCACCTACATCTGCAC	CAGGGCAGCTTCTGTCTTCT
IL1RN	GCCTGTGTCAAGTCTGGTGA	CCTCCTTGTTCTTGCTCAGG

### Immunocytochemistry

Cells were cultured on gelatin-coated (Sigma) coverslips, fixed in 3% paraformaldehyde for 20 min and permeabilised for 1 h with 0.1% triton-X-100. Blocking was carried out with 2.5% normal horse serum for 20 min. Incubation with primary antibodies was done at 4 °C overnight in blocking solution before washing and detection with secondary antibody. Primary antibodies used were 5 µg/ml mouse anti-NFκB (p65, 436700, ThermoFisher, Rockford, USA) and 2 µg/ml rabbit anti-IL1R1 (HPA029560, Sigma). Secondary antibodies used were 10 µg/ml goat anti-mouse IgG Alexaflor 594(A11005, Thermo Fisher Scientific) and 10 µg/ml goat anti-rabbit IgG Alexaflor 594 (A11012, Thermo Fisher Scientific). Negative controls were performed using secondary antibody alone. Coverslips were mounted using Vectashield Hardset containing DAPI (4′,6-diamidino-2-phenylindole, Vector Laboratories). Staining intensity in the nucleus was quantified using ImageJ.

### Ethics approval and consent to participate

Tissue samples were collected with the approval of the Animal Health Trust Research Ethics committee (AHT_02_2012) and all experiments were performed in accordance with relevant guidelines and regulations.

## Supplementary information

Supplementary information

## Data Availability

All data generated or analysed during this study are included in this published article.
