# Supplementary material for: A novel mechanism for the protection of embryonic stem cell derived tenocytes from inflammatory cytokine interleukin 1 beta
Source: Sci Rep. 2019 Feb 26;9:2755. doi: 10.1038/s41598-019-39370-4 (PMC6391488; doi:10.1038/s41598-019-39370-4)
Supplement: Supplementary file 1 — Supplementary information [file 41598_2019_39370_MOESM1_ESM.pdf]

A novel mechanism for the protection of embryonic stem cell derived tenocytes from inflammatory cytokine interleukin 1 beta

Alyce McClellan[1], Richard Evans[1], Cheryl Sze[1], Shohei Kan[1], Yasmin Paterson[1,2], Deborah Guest[1*]

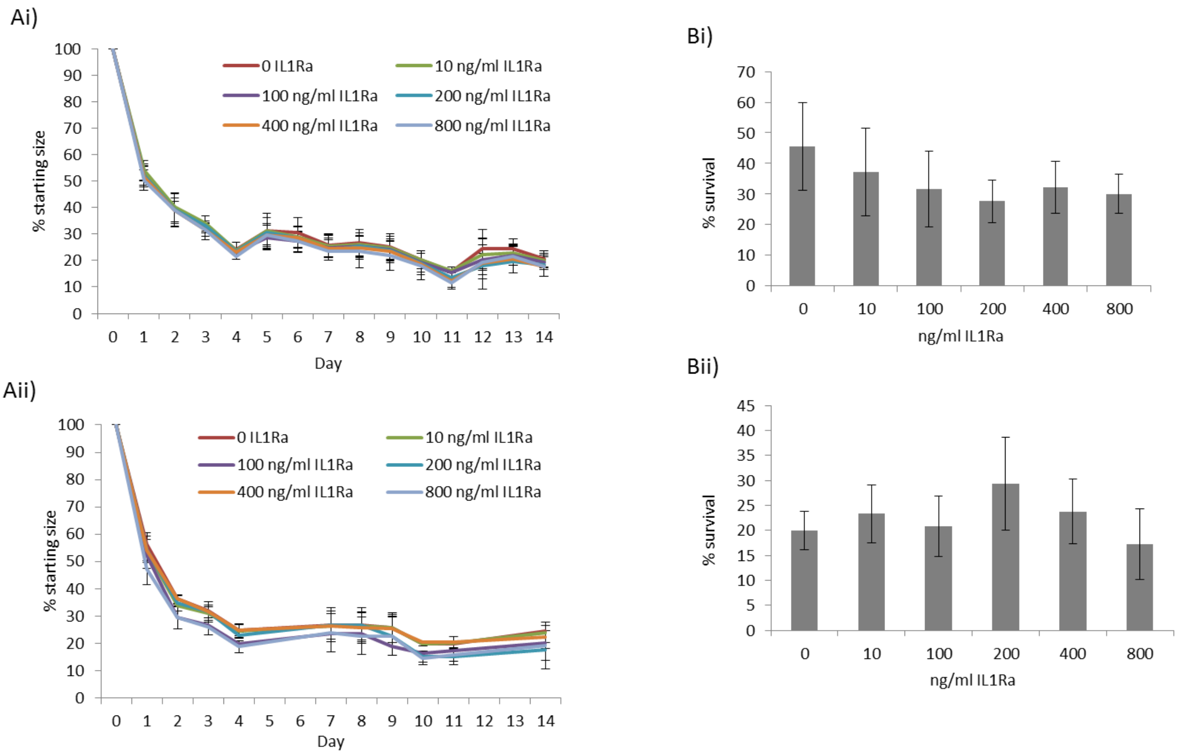

Supplementary Figure 1. In the absence of IL-1β IL1Ra alone has no effect on collagen gel contraction or tenocyte survival. A) The contraction of a collagen gel by (i) adult and (ii) fetal tenocytes is not affected by the presence of IL1Ra at any of the concentrations tested. B) The survival of (i) adult and (ii) fetal tenocytes in collagen gels after 14 days of culture is not affected by the presence of IL1Ra at any of the concentrations tested. Error bars represent the s.e.m of three independent biological replicates.
